# Non-Invasive Myocardial Work Detects Extensive Coronary Disease in Orthotopic Heart Transplant Patients

**DOI:** 10.3390/medsci13040212

**Published:** 2025-10-01

**Authors:** Rebeca Manrique Antón, Marina Pascual Izco, Agnés Díaz Dorronsoro, Ana Ezponda, Fátima de la Torre Carazo, Nahikari Salteráin, Leticia Jimeno-San Martín, Nerea Martín-Calvo, Áurea Manrique Antón, María Josefa Iribarren, Gorka Bastarrika, Gregorio Rábago

**Affiliations:** 1 Department of Cardiology and Cardiac Surgery, Clínica Universidad de Navarra, University of Navarra, Av. De Pío XII 36, 31007 Pamplona, Spain; mpascualiz@unav.es (M.P.I.); adiazdo@unav.es (A.D.D.); nsalterain@unav.es (N.S.); ljimeno@unav.es (L.J.-S.M.); grabago@unav.es (G.R.); 2 Instituto de Investigación Sanitaria de Navarra (IdiSNA), 31008 Pamplona, Spain; aezponda@unav.es (A.E.); bastarrika@unav.es (G.B.); 3 Department of Radiology, Clínica Universidad de Navarra, University of Navarra, Av. De Pío XII 36, 31007 Pamplona, Spain; 4 Department of Cardiology, Hospital Universitario Ramón y Cajal, 28034 Madrid, Spain; fdelatorrec@gmail.com; 5 Department of Preventive Medicine and Public Health, University of Navarra, 31008 Pamplona, Spain; nmartincalvo@unav.es; 6 The Hub FP. Calle Cádiz, 50004 Zaragoza, Spain; 7 Department of Anesthesia and Critical Care, Clínica Universidad de Navarra, University of Navarra, Av. De Pío XII 36, 31007 Pamplona, Spain; mjiriba@unav.es

**Keywords:** heart transplantation, extensive coronary disease, SIS, myocardial work

## Abstract

Background/Objectives: Cardiac allograft vasculopathy (CAV) remains a prevalent and serious long-term complication following orthotopic heart transplantation (OHT), contributing substantially to graft failure and patient mortality. Given the adverse prognostic impact of extensive coronary artery involvement, this study investigates whether myocardial work (MW) indices can serve as a non-invasive tool to detect OHT recipients with a high burden of coronary disease. Methods: In this prospective study, 55 OHT recipients underwent paired evaluations with coronary computed tomography angiography (CCTA) and transthoracic echocardiography (TTE) during routine follow-up. From the echocardiograms, global longitudinal strain (GLS) of the left ventricle (LV) and myocardial work (MW) indices were derived. Patients were classified into two groups according to CCTA findings: those without extensive coronary artery disease (disease affecting fewer than four coronary segments or none, OHT < 4) and those with extensive disease (disease of four or more coronary artery segments, OHT ≥ 4). Results: CCTA revealed extensive coronary disease in 38 OHT recipients, while 17 had involvement of fewer than four segments or none. Between-group comparisons showed significant differences in global wasted work (GWW, energy expended without generating forward flow) and global work efficiency (GWE, the percentage of constructive work relative to total work). Using the Youden Index, the optimal thresholds for identifying extensive disease were GWW > 88 mmHg% and GWE < 94%. Patients exceeding these thresholds had a markedly higher probability of having ≥ 4 affected segments, with ORs of 4.61 for pathological GWW and 3.68 for pathological GWE compared to those with normal values. Conclusions: GWW and GWE demonstrated the strongest performance for identifying OHT recipients with extensive coronary disease. If confirmed in larger cohorts, these indices could offer a practical, non-invasive approach for detecting extensive CAV.

## 1. Introduction

Cardiac allograft vasculopathy (CAV) is among the most frequent complications after orthotopic heart transplantation (OHT) and remains a major cause of graft failure and mortality [1]. Histologically, it is characterized by diffuse intimal thickening that compromises myocardial perfusion. Owing to the denervation of the transplanted heart, the disease often progresses silently [2,3,4,5]. For this reason, the International Society for Heart and Lung Transplantation (ISHLT) recommends annual or biennial coronary angiography, with or without intravascular ultrasound, for early detection [6]. However, these procedures have important drawbacks, including their invasive nature, associated costs, and the need for repeated examinations over time [7,8,9,10].

Coronary computed tomography angiography (CCTA) enables non-invasive visualization of CAV in epicardial vessels measuring ≥ 1.5 mm in diameter [11]. In the OHT population, it has proven valuable for stratifying long-term cardiovascular risk. Quantifying disease burden through the 16-segment model of the American Heart Association or the Segment Involvement Score (SIS) [12] provides key prognostic insight into the likelihood of major adverse cardiovascular events (MACEs), including myocardial infarction, stroke, need for coronary revascularization, or cardiovascular death. Notably, involvement of four or more coronary segments has been strongly linked to a higher incidence of MACEs [13].

Despite its diagnostic value, CCTA requires the use of iodinated contrast and exposes patients to ionizing radiation. These considerations are especially important in OHT recipients, who frequently present with reduced renal function and undergo repeated imaging over time, resulting in cumulative radiation exposure [14].

These limitations have driven interest in developing alternative methods for detecting CAV that are non-invasive, free from radiation, and do not require contrast administration. Advanced echocardiographic modalities have emerged as a promising option [14], with GLS demonstrating correlations with both the presence and severity of CAV [15,16]. Nevertheless, the diagnostic consistency of strain-based measures is hindered by their susceptibility to changes in loading conditions [17].

Within this search for improved non-invasive strategies, myocardial work (MW) may provide a useful approach for assessing left ventricular (LV) contractile performance through pressure–strain loop analysis. By integrating arterial pressure data with strain measurements, MW offers a more comprehensive assessment of myocardial function [17]. Initial studies have demonstrated its value for identifying coronary disease in the general population, and growing evidence supports its application in adult OHT recipients [17,18,19,20]. In particular, global work efficiency (GWE) has shown independent associations with CAV, highlighting its potential as a dependable non-invasive marker of disease burden [21,22].

The aim of this study is to evaluate the utility of MW in identifying OHT patients with extensive coronary disease, defined as disease of 4 or more segments according to the SIS scale.

## 2. Materials and Methods

### 2.1. Study Subjects

We conducted a prospective study involving 55 consecutive OHT recipients who underwent both CCTA and transthoracic echocardiography (TTE) during routine biannual follow-up visits at our institution between March 2021 and March 2023. Patients were enrolled consecutively in accordance with hospital protocol. TTE was generally performed within 2–3 h after CCTA, except in a few cases where the tests were scheduled on consecutive days. All images were evaluated by independent specialists blinded to the results of other investigations and to clinical data.

Patients were excluded if they had poor image quality—caused by arrhythmias during CCTA (n = 1) or inadequate acoustic windows on TTE (n = 2).

The study protocol was approved by the institutional Ethics Committee, adhered to the principles of the Declaration of Helsinki, and all participants provided written informed consent prior to enrollment.

### 2.2. Demographic and Clinical Data

Information collected included age, sex, body mass index (BMI), and cardiovascular risk factors (hypertension, hyperlipidemia, diabetes mellitus, and smoking status). Clinical parameters recorded were eGFR, hemoglobin concentration, prior cytomegalovirus infection, presence of implanted cardiac devices, and history of peripheral artery disease. Medication data were also compiled, covering immunosuppressive regimens, antiplatelet drugs, and lipid-lowering agents.

### 2.3. Two-Dimensional TTE

TTE was performed in all OHT recipients using a Vivid™ E95 4D platform equipped with an M5S 3.5-MHz transducer (GE Healthcare, Milwaukee, WI, USA), with subjects in the left lateral decubitus position. Standard 2D images were acquired and stored as digital cineloops for offline analysis.

From the parasternal view, measurements included LV septal thickness (IVSd), posterior wall thickness (PWd), and LV end-diastolic diameter (LVDd). LV mass index was obtained by dividing anatomic LV mass by body surface area, and relative wall thickness (RWT) was calculated as (2 × PWd)/(LVDd). From the apical view, LVEF was determined using the biplane disc method from end-systolic and end-diastolic volumes. Diastolic function assessment included measurement of peak early (E) and late (A) transmitral velocities, E/A ratio, E-wave deceleration time (DT), and pulsed wave Tissue Doppler velocities (E′) at septal and lateral mitral annuli. The E/E′ ratio was used to estimate LV filling pressure, and isovolumetric relaxation time (IRT) was also obtained. All image acquisition and Doppler measurements followed current European and American guideline recommendations [23].

**Figure 1:**
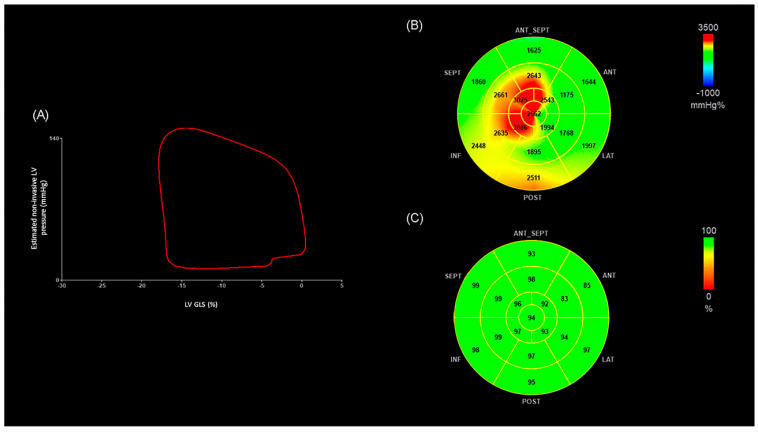
(**A**) Example of a non-invasive LV pressure–strain loop from an OHT patient without extensive coronary disease. The area inside the loop corresponds to the MWI. (**B**) Segmental MWI bull’s-eye plot for the same patient, showing normal values. (**C**) GWE bull’s-eye plot for the same patient, indicating normal efficiency. ANT: anterior; ANT_SEPT: anteroseptal; GWE: global work efficiency; INF: inferior; LAT: lateral; GLS: global longitudinal strain; LV: left ventricle; MWI: myocardial work index; OHT: orthotopic heart transplantation; POST: posterior; SEPT: septal.

For MW analysis, 2D images were first acquired from the apical four-, two-, and three-chamber views to obtain GLS using vendor-specific software (EchoPAC version 202; GE Healthcare, Milwaukee, WI, USA). The same application then merged GLS data with a non-invasively estimated LV pressure curve, constructed from brachial BP measurements and valve timing events identified on 2D echocardiography. This process generated a pressure–strain loop (Figure 1), from which four MW indices were derived. MWI reflected the total amount of mechanical work performed by the LV during systole, quantified as the area enclosed by the loop. GCW represented the fraction of that work that effectively contributed to blood ejection, whereas GWW described the energy expended without producing forward flow. GWE indicated the percentage of constructive work relative to total work, providing an overall measure of LV performance efficiency [17,24].

### 2.4. CCTA Protocol and Study Analysis

All CCTA studies were carried out using a 256-slice CT system (Revolution CT, GE Healthcare, USA). When patient rhythm and cooperation allowed, image acquisition was synchronized to the ECG in a prospective mode to help limit radiation exposure. Scanning was typically performed with a tube voltage between 100 and 120 kV and a rotation time of 0.28 s, while tube current was modulated according to body habitus. Images were reconstructed at a slice thickness of 0.625 mm. For contrast enhancement, 80–100 mL of iodinated contrast medium was administered at 4–5 mL/s, immediately followed by a 30–50 mL saline flush. If necessary, beta-blockers were used to reduce the heart rate below 65 bpm, and 0.4 mg of sublingual nitroglycerin was given just before image acquisition to improve coronary vasodilation. Coronary involvement was quantified using the 16-segment American Heart Association (AHA) [25] model, with SIS representing the number of segments containing any plaque, irrespective of stenosis severity. Extensive disease was defined as the presence of lesions in four or more segments.

### 2.5. Statistical Analysis

Continuous variables are presented as mean ± standard deviations (SD) or as median with interquartile range, depending on the distribution of the data. Categorical variables are shown as counts and percentages. Comparisons between groups were made using either the independent samples *t* test or the Mann–Whitney U test for continuous data, and the chi-square or Fisher’s exact test for categorical data. ROC curves were generated to identify cut-off values for echocardiographic variables, with the Youden Index applied to determine the point that best balanced sensitivity and specificity. Logistic regression was used to explore independent predictors of extensive coronary disease. Statistical significance was set at *p* < 0.05. All analyses were conducted using SPSS (version 25.0; IBM Corp., Armonk, NY, USA).

## 3. Results

A total of 55 consecutive OHT patients were recruited and underwent both CCTA and TTE. CCTA identified disease in four or more coronary artery segments in 38 OHT patients, while 17 had coronary disease in fewer than four segments or no disease at all.

Data from two groups were analyzed to identify echocardiographic variables that could distinguish patients with extensive coronary artery disease: OHT without extensive coronary artery disease (OHT < 4) and OHT patients with extensive coronary artery disease (OHT ≥ 4).

Demographic and clinical characteristics of the two groups are summarized in Table 1.

A higher prevalence of disease in ≥4 coronary segments was observed in male patients (*p* < 0.01) and in those with dilated cardiomyopathy as the primary etiology (*p* < 0.05). Additionally, the use of mycophenolate was more common in the OHT < 4 group, while everolimus use was significantly higher in the OHT ≥ 4 (both *p* < 0.01). Also, the time from OHT to CCTA was significantly longer in OHT ≥ 4 compared to those OHT < 4 (*p* < 0.05).

### Advanced Echocardiography Data

Advanced echocardiographic variables are summarised in Table 2.

In this analysis of advanced echocardiographic variables, only GWE exhibited a marginally significant difference between the two groups of heart transplant recipients (*p* = 0.05), being higher in the OHT < 4 group compared to those with OHT ≥ 4. On the other hand, although a trend was observed, differences between groups for variables such as GLS, MWI, GCW, and GWW did not reach statistical significance.

Table 3 presents the cutoff values for the echocardiographic variables identified using the Youden index.

Table 4 displays the percentage of patients with pathological values for each advanced echocardiographic variable, categorized according to the Youden index: OHT < 4 and OHT ≥ 4. GWW and GWE values differed significantly between patients with and without extensive coronary disease. Pathological GLS (*p* < 0.05), pathological GWW (*p* = 0.01), and pathological GWE (*p* < 0.05) were all significantly more prevalent in the OHT ≥ 4 group compared to the OHT < 4 group.

Table 5 presents the OR with 95% CI for obtaining a pathological result in each of the advanced echocardiographic variables associated with an increasing number of coronary segments affected. Each affected coronary segment represents a 22% relative increase in the risk of obtaining a pathological result in GLS (95% CI: 2–47%). Similarly, for each additional affected coronary segment, the likelihood of obtaining a pathological result in GWW increases by a factor of 1.20 (95% CI: 1.03–1.40).

Table 6 presents the OR with 95% CI for the presence of lesions in ≥4 coronary artery segments in OHT recipients, associated with each advanced echocardiographic variable.

Patients with abnormal GWW had an OR of 4.61 for the presence of ≥4 diseased segments, while those with abnormal GWE had an OR of 3.68, compared with individuals whose values were within the normal range.

Table 7 displays the sensitivity and specificity values calculated for each variable using the Youden index, along with the cutoff points applied for dichotomization and the ability of each parameter to correctly identify patients with OHT ≥ 4.

The best AUC results were observed for GWW and GWE.

**Figure 2:**
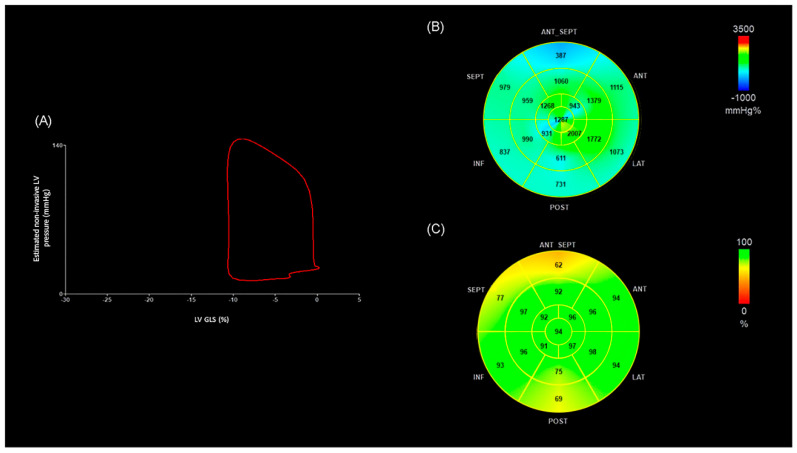
(**A**) Example of a non-invasive LV pressure–strain loop from an OHT patient with extensive coronary artery involvement, illustrating a smaller loop area compared to the patient in Figure 1. (**B**) Segmental MWI bull’s-eye view for the same patient, with blue sectors marking reduced values. (**C**) GWE bull’s-eye view for the same patient, highlighting multiple segments with impaired efficiency. ANT: anterior; ANT_SEPT: anteroseptal; GWE: global work efficiency; INF: inferior; LAT: lateral; GLS: global longitudinal strain; LV: left ventricle; MWI: myocardial work index; OHT: orthotopic heart transplantation; POST: posterior; SEPT: septal.

Figure 2 shows representative images of LV pressure–strain loop diagrams and segmental bull’s-eye GWE plots from an OHT patient with >4 pathological segments. The area of the LV pressure–strain loop is visibly smaller and the GWE values are lower compared with those in Figure 1. In addition, the MWI and GWE bull’s-eye plots illustrate multiple segments with reduced values, highlighted in blue, consistent with impaired efficiency.

## 4. Discussion

This study shows that MW—especially GWW and GWE—can identify OHT recipients with extensive coronary disease (SIS ≥ 4). Among the echocardiographic variables assessed, GWW and GWE provided the best discrimination, and pathological values of GWW and GWE were independently associated with extensive disease.

The diagnosis of extensive coronary disease in our cohort was established by CCTA, whose diagnostic accuracy against invasive angiography has been demonstrated in OHT recipients, with reported sensitivity and specificity of 95% and 79%, respectively [11].

The main goal of this work was to establish a non-invasive method for identifying patients with extensive coronary artery disease, regardless of their formal CAV grading [26]. According to the ISHLT consensus, CAV is classified based on both coronary anatomy and functional parameters such as LVEF ≤ 45% or restrictive physiology [14,27]. Nevertheless, the presence of extensive coronary artery disease itself—independent of the ISHLT grading—has been consistently associated with worse clinical outcomes in heart transplant recipients [13]. Although survival outcomes specifically stratified by the extent of coronary involvement (e.g., SIS ≥ 4) have not been reported, higher ISHLT angiographic grades of CAV are clearly associated with worse prognosis. In a large cohort, Van Keer et al. observed that patients with CAV 2–3 had a 5-year survival of 46% compared with 68% in those with CAV 0–1, and were more often retransplanted (12% vs. 0.5%) or died from a cardiac cause (22% vs. 5%). In multivariable analyses, the risk of death or retransplantation increased stepwise with CAV severity, with adjusted hazard ratios of 1.22 for CAV 1, 1.86 for CAV 2, and 5.71 for CAV 3 compared with patients without CAV [28]. These findings underscore the adverse prognostic impact of more severe coronary involvement, supporting the rationale for evaluating disease extent as in the present study.

The incorporation of advanced echocardiographic parameters such as MW into the follow-up of OHT recipients is supported by the European Association of Cardiovascular Imaging since 2015 [14]. Non-invasive MW has already demonstrated its value in detecting coronary artery disease in the general population [18,19,20] and, more specifically, in identifying obstructive coronary lesions in OHT recipients [21,22]. However, limited information exists regarding reference MW values for adult OHT recipients. In our study, patients without extensive disease (SIS < 4) still showed lower MW indices than those reported for individuals of similar age in the general population, suggesting that the transplantation process itself may impair myocardial performance [17].

Prior studies in pediatric and adult OHT populations have similarly shown impaired MW indices compared with healthy controls, even in the absence of angiographically evident CAV. In those cohorts, the presence of CAV was consistently associated with further deterioration in GWE and other functional parameters, reinforcing the potential role of MW as a screening tool [21,29].

Our previous work also highlighted GWE as the strongest echocardiographic predictor of obstructive coronary lesions in OHT recipients [22]. The present analysis extends those findings by demonstrating that GWE is not only associated with obstructive lesions, but also with the presence of extensive disease. In our cohort, patients with SIS ≥ 4 had reduced GWE and elevated GWW compared with those with less extensive involvement, reflecting greater impairment of LV systolic performance when multiple coronary territories are affected.

The prognostic importance of extensive coronary involvement is well established in both the general population and in transplant recipients. Several large studies have shown that patients with significant stenosis affecting multiple coronary segments experience a markedly higher incidence of major adverse cardiac events (MACE). In fact, the risk rises progressively with the number of diseased segments, underscoring the additive prognostic burden of diffuse coronary disease [30,31].

In the specific setting of OHT, previous work has confirmed that both the presence of ≥50% stenosis and extensive segmental involvement (SIS ≥ 4) are independently associated with a substantially higher rate of MACE [13]. These findings highlight the relevance of not only grading coronary lesions anatomically, but also considering the extent of vascular involvement when assessing long-term prognosis in heart transplant recipients.

In our series, pathological GWW and GWE values were strongly associated with extensive disease on CCTA. Specifically, the odds of having ≥ 4 diseased segments were more than fourfold higher in patients with abnormal GWW and nearly fourfold higher in those with abnormal GWE. Moreover, each additional affected segment was associated with a progressive increase in the likelihood of pathological myocardial mechanics. In addition, although GLS did not differ significantly in mean values between groups, pathological GLS values were more frequently observed among patients with extensive disease, and its high specificity suggests a potential role in ruling out CAV.

Taken together, our results indicate that MW parameters, particularly GWW and GWE, can provide valuable information for identifying OHT recipients with extensive coronary involvement, as defined by SIS ≥ 4. These indices offer a load-adjusted assessment of LV systolic function and may contribute to refining patient monitoring strategies after transplantation. While our findings support the potential role of MW in this setting, larger multicenter studies are needed to validate these observations, establish robust reference values for the OHT population, and determine whether MW-guided follow-up can translate into improved clinical outcomes.

Some limitations should be acknowledged. The small cohort size may compromise statistical power and limit generalizability. Variability in MW values may also arise from the heterogeneous population, which included both recently transplanted patients and long-term survivors. Potential structural changes of the graft, such as scar formation or diffuse fibrosis secondary to prior rejection episodes, were not evaluated in our cohort. This factor may have had an impact on the echocardiographic analysis and should be considered when interpreting our findings. Moreover, the cross-sectional nature of the study does not allow monitoring of myocardial work dynamics as coronary lesions progress over time.

## 5. Conclusions

We report here the first study specifically designed to investigate myocardial work for detecting extensive coronary artery disease in OHT patients. GWW and GWE demonstrated the strongest associations with disease presence. Should these findings be replicated in larger studies, MW could become a readily available, non-invasive approach to identify patients at increased prognostic risk.

## Figures and Tables

**Table 1:** Demographic and clinical characteristics of OHT < 4 and OHT ≥ 4 patients.

	OHT < 4 (n = 17)	OHT ≥ 4 (n = 38)	*p*
Age (years)	60.43 (13.94)	64.68 (11.51)	0.27
Sex (male)	8 (47.06)	34 (89.47)	<0.01
BMI (kg/m^2^)	27.44 (6.26)	25.45 (3.42)	0.39
SBP (mmHg)	122.94 (17.18)	127.58 (20.08)	0.48
DBP (mmHg)	82.24 (13.86)	79.63 (12.50)	0.55
Hypertension	11 (64.71)	24 (63.16)	1
Dyslipidemia	11 (64.71)	28 (73.68)	0.53
Diabetes mellitus	2 (11.76)	11 (28.95)	0.30
Tobacco use Never Current Former	23 (48.94) 2 (4.26) 22(46.81)	1 (14.29) 0 (0.00) 6 (85.71)	0.89
Age at OHT (years)	53.24 (13.97)	53.32 (12.08)	0.73
Retransplant	1 (5.88)	0 (0.00)	0.30
eGFR (mL/min/1.73 m^2^)	55.12 (9.88)	56.26 (7.50)	0.77
Hemoglobin (g/dL)	14.38 (2.85)	13.60 (2.01)	0.57
Cytomegalovirus infection	8 (47.06)	16 (42.11)	0.77
Cardiac device	0 (0.00)	2 (5.26)	1
Peripheral artery disease	3 (17.65)	3 (7.89)	0.35
Etiology of the cardiopathy: Dilated Ischemic Valvular Congenital CAV Autoimmune	3 (17.65) 8 (47.06) 1 (5.88) 3 (17.65) 1 (5.88) 1 (5.88)	20 (52.63) 13 (34.21) 4 (10.53) 1 (2.63) 0 (0.00) 0 (0.00)	<0.05 0.38 1 0.08 0.30 0.30
Antiplatelet: Acetylsalicylic acid Clopidogrel Ticlopidine	15 (88.24) 1 (5.88) 1 (5.88)	28 (73.68) 7 (18.42) 4 (10.53)	0.30 0.41 1
Immunosuppressive agents: Tacrolimus Mycophenolate mofetil Everolimus Steroids Cyclosporine Sirolimus	17 (100.00) 17 (100.00) 0 (0.00) 3 (17.65) 0 (0.00) 0 (0.00)	35 (92.11) 25 (65.79) 13 (34.21) 5 (13.16) 2 (5.26) 1 (2.63)	0.54 <0.01 <0.01 0.69 1 1
Lipid-lowering drugs: Statin Ezetimib	14 (82.35) 3 (17.65)	34 (89.47) 7 (18.42)	0.664 1
Time from OHT to CCTA (months)	62.93 (37.43–129.70)	94.70 (68.46–198.27)	0.028

Data are reported as means and standard deviations for normally distributed variables, and as median and interquartile ranges for non-normally distributed variables. DBP: diastolic blood pressure; BMI: body mass index; CAV: coronary allograft vasculopathy; CCTA: coronary computed tomography angiography; eGFR: estimated glomerular filtration rate; OHT: orthotopic heart transplant recipients; OHT < 4: orthotopic heart transplant recipients without extensive coronary artery disease on coronary computed tomography angiography; OHT ≥ 4: orthotopic heart transplant recipients with extensive coronary artery disease on coronary computed tomography angiography; SBP: systolic blood pressure.

**Table 2:** Advanced echocardiographic parameters in HV, OHT < 4 and OHT ≥ 4 patients.

		OHT < 4 (n = 17)	OHT ≥ 4 (n = 38)	*p*
GLS (%)	Mean	−15.47 (2.28)	−2.28 (5.83)	0.27
	p50	−16	−15	
	p25	−17	−16	
	p75	−14	−11	
MWI (mmHg%)	Mean	1419.77 (373.91)	1391.55 (380.38)	0.87
	p50	1406	1374	
	p25	1213	1145	
	p75	1538	1663	
GCW (mmHg%)	Mean	1637.12 (377.06)	1645 (446.05)	0.66
	p50	1573	1713	
	p25	1387	1360	
	p75	1767	1934	
GWW (mmHg%)	Mean	96.59 (82.91)	111.42 (66.27)	0.08
	p50	78	91	
	p25	52	73	
	p75	95	132	
GWE (%)	Mean	93.18 (5.45)	91.42 (4.64)	0.05
	p50	95	92	
	p25	93	88	
	p75	96	95	

Means and standard deviations are presented with two decimal places to reflect estimation precision, while percentiles (p25, p50, p75) are rounded to whole numbers for easier interpretation. GCW: global constructive work; GLS: global longitudinal strain of left ventricle; GWE: global work efficiency; GWW: global wasted work; mmHg: millimeters of mercury; MWI: myocardial work index.

**Table 3:** Cutoff points for advanced echocardiographic variables calculated using the Youden index.

	Cutoff
GLS	>−12.6
MWI	<1131
GCW	<1767
GWW	>88
GWE	<94

GCW: global constructive work; GLS: global longitudinal strain of left ventricle; GWE: global work efficiency; GWW: global wasted work; MWI: myocardial work index.

**Table 4:** Percentage of patients with pathological values in advanced echocardiographic variables according to the Youden index.

Pathological Values (%)	OHT < 4 (n = 17)	OHT ≥ 4 (n = 38)	*p*
GLS	5.88	31.58	0.04
MWI	11.76	23.68	0.31
GCW	70.59	55.26	0.28
GWW	29.41	65.79	0.01
GWE	29.41	60.53	0.03

GCW: global constructive work; GLS: global longitudinal strain of left ventricle; GWE: global work efficiency; GWW: global wasted work; MWI: myocardial work index. OHT < 4: orthotopic heart transplant recipients without extensive coronary artery disease on coronary computed tomography angiography; OHT ≥ 4: orthotopic heart transplant recipients with extensive coronary artery disease on coronary computed tomography angiography.

**Table 5:** Odds ratios (OR) for each pathological segment.

Pathological Values	OR (CI 95%)	*p*
GLS	1.22 (1.02–1.47)	0.033
MWI	1.14 (0.95–1.40)	0.152
GCW	0.92 (0.79–1.06)	0.236
GWW	1.20 (1.03–1.40)	0.018
**GWE**	1.10 (0.96–1.27)	0.175

CI: confidence interval; GCW: global constructive work; GLS: global longitudinal strain; GWE: global work efficiency; GWW: global wasted work; MWI: myocardial work index. OR: odds ratio.

**Table 6:** OR for the presence of ≥4 pathological segments.

Pathological Values	OR (CI 95%)	*p*
GLS	7.38 (0.87–62.32)	0.07
MWI	2.32 (0.44–12.17)	0.32
GCW	0.51 (0.15–1.75)	0.24
GWW	4.61 (1.33–15.95)	0.02
GWE	3.68 (1.07–12.58)	0.04

CI: confidence interval; GCW: global constructive work; GLS: global longitudinal strain; GWE: global work efficiency; GWW: global wasted work; MWI: myocardial work index; OR: odds ratio.

**Table 7:** Cutoff points according to the Youden index, sensitivity, specificity, and AUC for OHT ≥ 4 coronary segments affected.

	Cutoff Value	AUC (CI 95%)	Sensitivity	Specificity
GLS	−12.6	0.59 (0.43–0.74)	31.58	94.12
MWI	1131	0.48 (0.32–0.65)	88.24	23.68
GCW	1767	0.46 (0.29–0.63)	29.41	55.26
GWW	88	0.64 (0.47–81.3)	65.79	70.59
GWE	94	0.61 (0.45–0.77)	70.59	60.53

AUC: area under the curve; CI: confidence interval; GCW: global constructive work; GLS: global longitudinal strain; GWE: global work efficiency; GWW: global wasted work; MWI: myocardial work index.

## Data Availability

The data underlying this study are available from the corresponding author upon justified request. Because of ethical and privacy restrictions related to participant confidentiality, the datasets are not openly accessible. Access will be considered for qualified researchers, subject to relevant data protection regulations.

## Abbreviations

The following abbreviations are used in this manuscript:

A wave	late diastolic mitral inflow velocity
ANT	Anterior
ANT_SEPT	Anteroseptal
AUC	area under the curve
BMI	body mass index
CAV	cardiac allograft vasculopathy
CCTA	coronary computed tomography angiography
CI	confidence interval
CO	cardiac output
DBP	diastolic blood pressure
eGFR	estimated glomerular filtration rate
GCW	global constructed work
GWE	global work efficiency
GWW	global wasted work
ICA	invasive coronary angiography
INF	Inferior
ISHLT	International Society for Heart and Lung Transplantation
IVUS	intravascular ultrasound
LAT	Lateral
LV	left ventricle, left ventricular
LVEF	left ventricular ejection fraction
GLS	left ventricular global longitudinal strain
mmHg	millimeters of mercury
MW	myocardial work
MWI	myocardial work index
NPV	negative predictive value
OCL	obstructive coronary lesions
OHT	orthotopic heart transplantation
OHT< 4	orthotopic heart transplantation patients with coronary artery disease of fewer than 4 segments or no disease on CCTA
OHT ≥ 4	orthotopic heart transplantation patients with disease of 4 or more coronary artery segments on CCTA
OR	odds ratio
POST	Posterior
PRA	panel reactive antibodies
ROC	receiver operating characteristic
SBP	systolic blood pressure
SEPT	Septal
TTE	transthoracic echocardiography
